# Proteomics Analysis of Monocyte-Derived Hepatocyte-Like Cells Identifies Integrin Beta 3 as a Specific Biomarker for Drug-Induced Liver Injury by Diclofenac

**DOI:** 10.3389/fphar.2018.00699

**Published:** 2018-07-04

**Authors:** Diana Dragoi, Andreas Benesic, Garwin Pichler, Nils A. Kulak, Harald S. Bartsch, Alexander L. Gerbes

**Affiliations:** ^1^Department of Medicine 2, Liver Centre Munich, University Hospital Munich, Munich, Germany; ^2^MetaHeps GmbH, Martinsried, Germany; ^3^Department of Proteomics and Signal Transduction, Max Planck Institute of Biochemistry, Martinsried, Germany; ^4^PreOmics GmbH, Martinsried, Germany; ^5^Institute of Pathology, Medical School, Ludwig Maximilian University, Munich, Germany

**Keywords:** DILI, biomarker, proteomics, drug-development, monocyte-derived hepatocyte-like cells

## Abstract

Idiosyncratic drug-induced liver injury (iDILI) is a major cause of acute liver failure resulting in liver transplantation or death. Prediction and diagnosis of iDILI remain a great challenge, as current models provide unsatisfying results in terms of sensitivity, specificity, and prognostic value. The absence of appropriate tools for iDILI detection also impairs the development of reliable biomarkers. Here, we report on a new method for identification of drug-specific biomarkers. We combined the advantages of monocyte-derived hepatocyte-like (MH) cells, able to mimic individual characteristics, with those of a novel mass spectrometry-based proteomics technology to assess potential biomarkers for Diclofenac-induced DILI. We found over 2,700 proteins differentially regulated in MH cells derived from individual patients. Herefrom, we identified integrin beta 3 (ITGB3) to be specifically upregulated in Diclofenac-treated MH cells from Diclofenac-DILI patients compared to control groups. Finally, we validated ITGB3 by flow cytometry analysis of whole blood and histological staining of liver biopsies derived from patients diagnosed with Diclofenac-DILI. In summary, our results show that biomarker candidates can be identified by proteomics analysis of MH cells. Application of this method to a broader range of drugs in the future will exploit its full potential for the development of drug-specific biomarkers. Data are available via ProteomeXchange with identifier PXD008918.

## Introduction

Drug-induced liver injury is the leading cause for acute liver failure in United States and Europe (Kullak-Ublick et al., 2017). Toxic reactions due to drug intake can be dose-dependent, as in the case of APAP ingestion and are termed “intrinsic DILI.” In contrast, iDILI affects only susceptible patients, and even though it is a rare event (1:10,000 to 1:100,000 per drug), it often results in death or liver transplantation (Bell and Chalasani, 2009). Preclinical models usually fail to predict iDILI (Atienzar et al., 2016) and thus, iDILI is often being detected only after market approval. Even then, iDILI diagnosis remains a diagnosis of exclusion after elimination of other etiologies for liver damage (e.g., viral infections such as hepatitis or autoimmune diseases). Consequently, iDILI diagnosis is incorrect in up to 25% of the cases (Teschke et al., 2014). Moreover, current causality assessment methods, such as the RUCAM score and expert opinion encounter difficulties in identifying the causative drug in polymedicated patients (Andrade et al., 2009; Anderson and Borlak, 2011; Teschke and Eickhoff, 2016). While both methods have their strengths, they both heavily rely on a comprehensive collection of data requiring extensive investigations and documentation of the course of disease (Danan and Teschke, 2016). Additionally, both methods require an experienced investigator to perform optimally. Since DILI is still a diagnosis of exclusion, correct causality assessment is time consuming and expensive (Hayashi, 2016).

The lack of a reliable *in vitro* diagnostic tool for iDILI also impairs the development of safety biomarkers, as false positive or false negative diagnosis impair the identification of specific biomarkers (Teschke et al., 2017). Thus, several attempts to develop safety biomarkers for iDILI provided unsatisfying results because of low predictive values (Singer et al., 2010; Spraggs et al., 2011). Since iDILI is a rare event, this may in part results from dilution caused by false positives in the suspected iDILI groups. Several promising biomarker candidates have recently been proposed, e.g., HMGB1, MCSFR1, cytokeratin 18, and micro-RNAs miR-122 and miR-192 (Robles-Díaz et al., 2016). However, some limitations exist: the majority of these markers are not liver specific (HMGB1, cytokeratin 18, and MCSFR1), and even if they specifically detect liver damage, the issue of causality remains the most pressing challenge. Moreover, none of these candidates has been shown to be drug-specific, which would be an important advantage in improving drug safety (Andrade et al., 2009). Thus, there is currently no valid diagnostic or prognostic biomarker available for iDILI (Teschke et al., 2017).

We have previously reported on the development of the first *in vitro* test able to diagnose iDILI using a blood sample from respective patients with iDILI suspicion. The test is based on the generation of MH cells, which reflect individual donor characteristics, such as toxic reactions to drug treatment (Benesic et al., 2012). This test is able to distinguish iDILI from other causes of liver damage and furthermore, to identify the culprit drug in polymedicated patients (Benesic et al., 2016, 2018; Benesic and Gerbes, 2018). In the current study, we combine the ability of MH cells to simulate specific toxic reactions in samples from individual donors with a new and improved proteomics technology in order to identify drug-specific biomarkers for iDILI (Kulak et al., 2014). Here, we focus on Diclofenac-DILI, since Diclofenac is the worldwide most prescribed NSAID (Altman et al., 2015) and is the most frequently implicated agent in DILI caused by NSAIDs (Fontana et al., 2009; Björnsson, 2016; Schmeltzer et al., 2016). Diclofenac-DILI displays a well characterized drug signature which allows accurate diagnosis by clinical assessment methods such as RUCAM (Boelsterli, 2003). Moreover, the injury mechanisms of Diclofenac are well described, involving both metabolic cell pathways and immunologic reactions (Boelsterli, 2003). Thus, our goal was to investigate whether Diclofenac-treatment of MH cells generated from patients clinically diagnosed with Diclofenac-DILI results in different protein expression patterns compared to vehicle control and to other control groups, respectively.

## Materials and Methods

### Patients and Causality Assessment

Subjects without liver damage and patients presenting with ALI and iDILI suspicion were included in this study. DILI was diagnosed by clinical causality assessment, according to previously published criteria (Benesic et al., 2016; see also Supplementary Table S1) as well as RUCAM (Danan and Teschke, 2016). The resulting classifications were “definite,” “highly likely,” or “probable” for iDILI and “possible” or “unlikely” for other ALI (liver injury due to other causes than iDILI). Informations on subjects and patients are presented in Supplementary Tables S2, S3.

### Cell Culture

Monocyte-derived hepatocyte-like cells were generated by gradient centrifugation and adherence separation of peripheral blood monocytes and cultured as described in Benesic et al. (2012). For toxicity testing, freshly generated MH cells were treated with 100 μM Diclofenac for 48 h (optimal concentration was determined by dose–response curves in 25 donors – unpublished data), followed by toxicity measurements, as described elsewhere (Benesic et al., 2016). In brief, following cell exposure to Diclofenac or vehicle, lactate dehydrogenase (LDH) was measured by optical density using the Promega Cytotox non-radioactive Assay^®^ (Promega, Mannheim, Germany) from supernatants and cell lysates after lysis with 0.1% TWEEN in MOPS buffered saline. Each condition was tested in triplicate. LDH release was calculated from ODsupernatant: (ODsupernatant + ODlysate). MH cell test results were considered as positive when increase in LDH-release exceeded the fourfold standard deviation of vehicle control. For proteomics analysis, cryoconserved MH cells were thawed and treated with 100 μM Diclofenac or vehicle (DMSO) for 48 h, followed by sample preparation for proteomics analysis.

### Proteomics Analysis

Sample preparation was performed as described previously (Kulak et al., 2014). For each condition, three biological replicates were used. Briefly, 50,000 MH cells were lysed, denatured, reduced, and alkylated in SDC buffer for 10 min at 95°C. Proteins were digested with Trypsin and LysC (1:100 w/w) at 37°C for 3 h. Acidified peptides were purified using SDB-RPS StageTips. Eluted peptides were completely dried using a SpeedVac centrifuge and suspended in LC loading buffer. Peptides were separated on a reverse phase column (50 cm length, 75 μm inner diameter) packed in-house with ReproSil-Pur C18-AQ 1.9 μm resin (Dr. Maisch GmbH, Ammerbuch, Germany). Reverse-phase chromatography was performed with an EASY-nLC 1000 ultrahigh pressure system, coupled to a Q-Exactive HF Mass Spectrometer (Thermo Scientific). Peptides were loaded with buffer A (0.1% (v/v) formic acid) and eluted with a nonlinear 100-min gradient of 5–60% buffer B (0.1% (v/v) formic acid, 80% (v/v) acetonitrile) at a flow rate of 250 nL/min. After each gradient, the column was washed with 95% buffer B and re-equilibrated with buffer A. Column temperature was kept at 50°C by an in-house designed oven with a Peltier element. Operational parameters were real-time monitored by the SprayQC software (Scheltema and Mann, 2012). Raw files were analyzed by MaxQuant software (version 1.5.3.2) (Cox and Mann, 2008) and peak lists were searched against the *Homo sapiens* Uniprot FASTA database (Version 2014/4) and a common contaminants database (247 entries) by the Andromeda search engine (Cox et al., 2011). Label-free quantification was done using the MaxLFQ algorithm (Cox et al., 2014) integrated into MaxQuant. Data was analyzed by using the Perseus software (Tyanova et al., 2016). Positions containing non-valid values were filtered out in order to obtain a protein data set present in all samples. Means and standard deviations were calculated from the remaining data and groups according to the study subjects were annotated. In addition, the annotations contained information on vehicle (baseline) or Diclofenac treatment (treated) of the respective MH cells. The data were log2-transformed and normalized using the *Z*-score function of the Perseus software. The MS proteomics data have been deposited to the ProteomeXchange Consortium via the PRIDE (Kullak-Ublick et al., 2017) partner repository with the data set identifier PXD008918.

### Flow Cytometry Analysis

Ethylenediaminetetraacetic acid-blood was diluted 1:2 in PBS 0.5% BSA and stained with 5 μL of anti-ITGB3 (#564174; BD Biosciences) or anti-IgG (#554681; BD Biosciences) for 45 min on ice. Samples were then washed twice, followed by erythrocyte lysis (lysis solution: 1.5 M NH_4_Cl, 0.1 M KHCO_3_, 1 mM EDTA disodium salt, in double distilled water) for 7–10 min at room temperature. Samples were then centrifuged, the cell pellet was resuspended in PBS 0.5% BSA and analyzed for ITGB3 expression using a BD Accuri flow cytometer (BD Biosciences).

### Immunohistochemistry

Liver samples for immunohistochemical analyses were selected from the tissue archives of the Institute of Pathology, Ludwig Maximilian University of Munich, Germany. Liver samples originated from patients with clinical indication for liver biopsy. Tissue sections were stained with anti-ITGB3 (Ventana) on an automated slide staining platform from Ventana, according to manufacturer’s instructions. For analysis, representative brightfield images were taken on a Leica DMD 108 microscope at the indicated magnifications. ITGB3-positive infiltration spots at inflammation sites were counted at 40× magnification.

### Statistics

Data analysis and statistics for proteomics data including PCA, Heat Map, hierarchical clustering were performed by Perseus software (Tyanova et al., 2016). The “proteinGroups.txt” file produced by MaxQuant was further analyzed in Perseus (version 1.5.1.6). PCA was done on the logarithmized LFQ intensities of all single-shot runs. Unsupervised hierarchical clustering of significantly (ANOVA, FDR < 0.05) regulated proteins (*z*-scored MaxLFQ values) was performed in Perseus. Data from FACS-analysis and immunohistochemistry were analyzed using SPSS-software (IBM SPSS Statistics 25.0). After testing for normal distribution Student’s *t*-test or Kruskal–Wallis test were applied. Where applicable the Chi-Square test was applied. Results were considered significant when *p* < 0.05.

## Results

**FIGURE 1:**
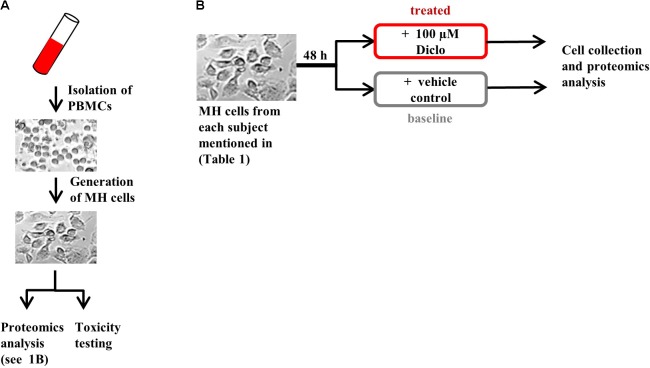
Experimental design. **(A)** Generation of MH cells followed by proteomics analysis and toxicity testing in parallel and **(B)** protocol for MH cell treatment prior to proteomics analysis. Brightfield images are shown for illustrative purposes only.

We included in our study six healthy subjects and 16 patients presenting with liver injury, initially suspected of iDILI. We further divided these subjects into two groups: one that had previously ingested Diclofenac (Diclo) and one which had never been exposed to Diclofenac before (Control), as a negative control (**Table 1**). Patients with liver injury were diagnosed based on clinical causality assessment (Supplementary Tables S1, S2) including RUCAM. This resulted in the following subgroups: patients suffering from iDILI due to Diclofenac (DicloDILI), patients suffering from iDILI due to other drugs (Diclo_otherDILI and Control_otherDILI) and patients suffering from liver injury due to other causes than iDILI (Diclo_otherALI and Control_otherALI) (**Figure 1A** and Supplementary Table S2). Subjects without liver injury were named Diclo_tolerator if they have been exposed to Diclofenac without resulting liver injury and Control_healthy if there was no Diclofenac exposure in the history. Importantly, all DicloDILI patients had a causality likelihood of at least “highly likely” for iDILI due to Diclofenac. In parallel to causality assessment, we generated MH cells from blood samples of each subject and used them for toxicity testing (MH cell test) and proteomics analysis (**Figures 1B,C**). The MH cell test was positive for Diclofenac only in DicloDILI patients, whereas co-medications in these patients tested negative (Supplementary Table S2). These results confirmed the previously reported ability of MH cells to mirror individual drug response (Benesic et al., 2016).

**Table 1:** Study subjects.

Diclofenac exposure	Yes	No	Comment
DILI due to Diclofenac	4 (DicloDILI)	n.a.	Patients with DILI due to Diclofenac
Healthy	3 (Diclo_tolerator)	3 (Control_healthy)	Subjects without signs of liver injury
DILI due to another drug	2 (Diclo_otherDILI)	4 (Control_otherDILI)	Patients with DILI due to another drug
Other ALI	3 (Diclo_otherALI)	3 (Control_otherALI)	Patients with acute liver injury of non-drug cause
Total	12	10	Total subjects: 22

*MH cells for proteomics analysis were obtained from 22 subjects: 12 patients exposed to Diclofenac of whom four had DILI due to Diclofenac (DicloDILI), three had tolerated Diclofenac without signs of liver injury (Diclo_tolerator), two had DILI due to another drug but were concomitantly treated with Diclofenac (Diclo_otherDILI), and three were exposed to Diclofenac but had acute liver injury due to a non-drug cause (Diclo_otherALI). Another 10 patients were never exposed to Diclofenac, of whom three had no signs of liver injury (Control_healthy), four had DILI due to a drug other than Diclofenac (Control_otherDILI), and three had acute liver injury due to a non-drug cause (Control_otherALI).*

**FIGURE 2:**
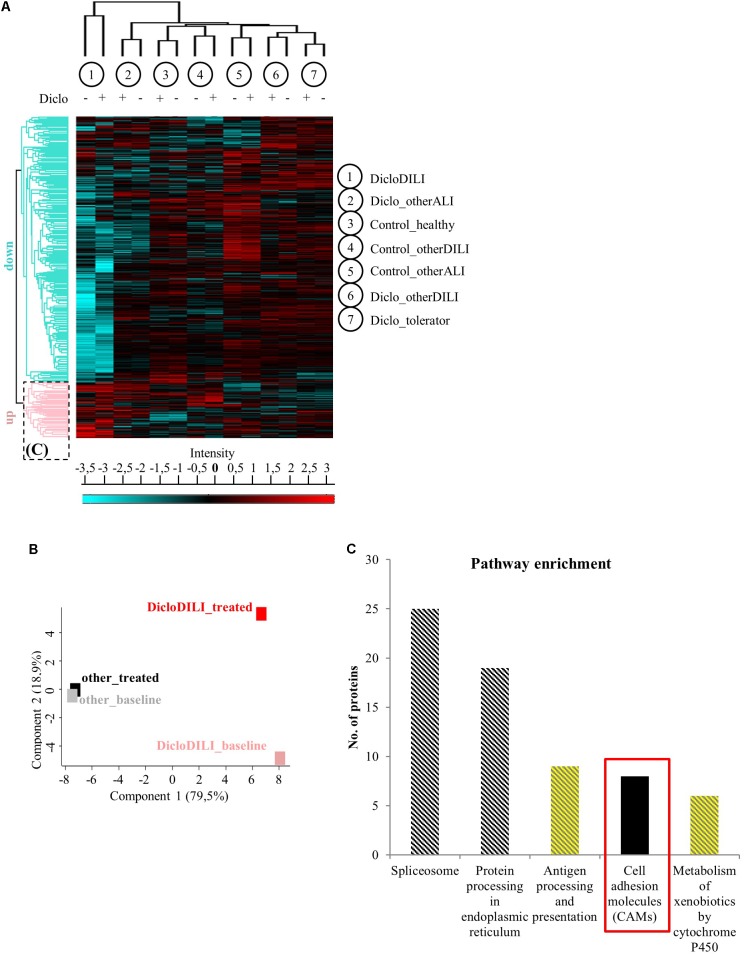
Proteomics analysis of MH cells identifies differentially regulated proteins in the investigated subject groups. **(A)** Hierarchical clustering of the different sample groups, treated with vehicle (–) or Diclofenac (+). Vertical clustering displays similarities between sample groups, while horizontal clusters reveal differentially regulated protein groups. The upregulated protein cluster marked by the *dotted rectangle* was further assessed for pathway enrichment, as displayed in **(C)**. **(B)** Principal component analysis of protein expression in MH cells treated with either vehicle (baseline) or 100 μM Diclofenac (treated). MH cells of DicloDILI patients were compared to all other groups (Diclo_tolerator, Diclo_otherALI, Diclo_otherDILI, Control_healthy, Control_otherALI, and Control_otherDILI), grouped under the term “other.” **(C)** Pathway enrichment analysis within the protein cluster mainly upregulated in DicloDILI (*dotted rectangle* from **A**).

Mass spectrometry-based proteomics analysis of MH cells from the individual patients identified more than 2,700 proteins expressed in all subject groups. Hierarchical clustering of this data set revealed considerable differences in protein expression between sample groups (**Figures 2A,B** and Supplementary Figure S1). As shown by the heat map diagram, DicloDILI specimens built a separate cluster compared to the other groups, independently of Diclofenac-treatment (vertical clustering in **Figure 2A**). Thus DicloDILI specimens clustered furthest away from samples of healthy/exposed subjects, which had received and tolerated Diclofenac. These observations were confirmed by PCA, showing that the proteome of untreated MH cells from DicloDILI patients (DicloDILI_baseline) display a unique signature compared to all other subject groups (termed “other_baseline”) (**Figure 2B** and Supplementary Figure S1). Moreover, protein expression in MH cells from DicloDILI patients showed a distinct shift after Diclofenac-treatment compared to vehicle control (DicloDILI_treated). In contrast, Diclofenac-treatment failed to induce substantial changes to protein expression in MH cells derived from nonDicloDILI subjects (other_treated; **Figure 2B**). Furthermore, hierarchical clustering identified two main protein expression clusters (horizontal clustering in **Figure 2A**): one that is predominantly downregulated in DicloDILI (turquois) and one that is mainly upregulated in DicloDILI (red). This distinct regulation pattern was only present in DicloDILI specimens and not in the other groups. Together with the results of the PCA analysis, this suggests that differences in the proteome of MH cells could reflect individual susceptibility to Diclofenac-DILI.

Since Diclofenac caused toxic responses only in MH cells of patients with Diclofenac-DILI and downregulation of proteins may be due to cytotoxicity, we focused on the protein cluster predominantly upregulated in DicloDILI (rectangle in **Figure 2A**) as a source of potential drug specific biomarkers. Therefore, we performed pathway enrichment analysis to reveal main biological mechanisms underlying DILI by Diclofenac (**Figure 2C**). Thus, we identified five signaling pathways, which were significantly upregulated in DicloDILI upon Diclofenac exposure (**Figure 2C** and Supplementary Table S4).

In order to identify specific biomarkers for Diclofenac-DILI, we searched for proteins that were upregulated upon Diclofenac-treatment exclusively in MH cells from DicloDILI patients and not in the other groups. Additionally, the potential biomarker should be easy to detect, e.g., from a blood sample, to facilitate clinical application. Under the top 10 upregulated proteins in MH cells from DicloDILI patients, only one membrane protein met these criteria: ITGB3, which was fourfold upregulated in the DicloDILI condition upon Diclofenac-treatment, in contrast to the other groups (Supplementary Table S5). In line with this finding, several cell adhesion molecules were also enriched within the protein cluster upregulated in DicloDILI (**Figure 2C**). ITGB3 is expressed on peripheral blood cells (Luscinskas and Lawler, 1994; Harris et al., 2000), which indicated that it can be measured in blood samples.

**FIGURE 3:**
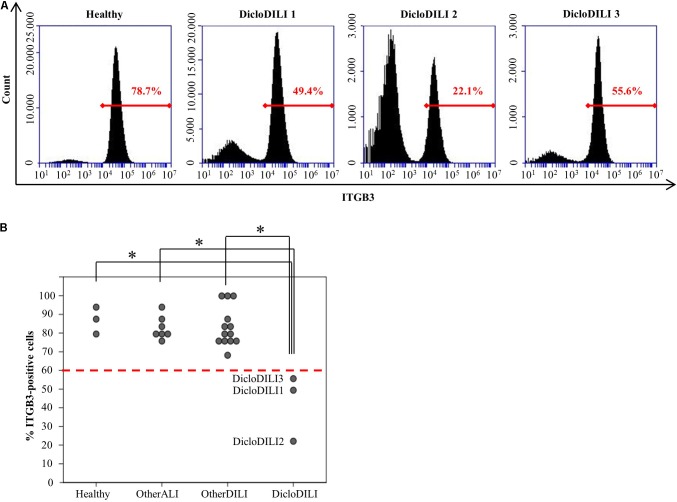
Diclofenac-DILI patients express lower levels of ITGB3 compared to control subjects. **(A)** Histogram plots of erythrocyte-lysed whole blood samples from three DicloDILI patients and one healthy subject, analyzed by flow cytometry for ITGB3 expression. In *red*: percentage of ITGB3-positive cells. **(B)** Distribution of ITGB3 values among subjects from each group. *n* = 3 for healthy, *n* = 7 for other ALI, *n* = 13 for other DILI, and *n* = 3 for DicloDILI. *Red dotted line*: threshold (= 60%) determined by receiver operating characteristic (ROC) analysis and used to discriminate between ITGB3-positive and ITGB3-negative samples. ^∗^*p* < 0.05.

Integrin beta 3 expression was determined by flow cytometry analysis of erythrocyte depleted whole blood (Supplementary Figure S2). Flow cytometry analysis of ITGB3 expression in the different subject groups revealed that patients diagnosed with Diclofenac-DILI displayed a reduction in the relative amount of ITGB3-positive cells in whole blood compared to other groups (**Figures 3A,B**). Based on the results of the ROC analysis, we determined a threshold of 60% ITGB3-positive cells in order to discriminate DicloDILI patients from other subjects. Importantly, all values of DicloDILI patients were below this threshold, while those of the other ALI and other DILI subjects were above the threshold. We also observed a correlation between the levels of ITGB3-positive cells and the course of liver parameters (Supplementary Figure S3). Thus, DicloDILI2 displayed the lowest relative amount of ITGB3-positive cells, and had the worst outcome, requiring liver transplantation. By contrast, DicloDILI3 yielded the highest value for ITGB3 among the DicloDILI group and recovered within a few weeks after the initial liver injury and stopping of Diclofenac administration.

We further investigated whether ITGB3 expression changes with disease progression/remission. DicloDILI1 and DicloDILI2 were included in the study during the acute phase of the drug reaction, also confirmed by strong elevation of the liver enzymes ALT and AST (Supplementary Figures S3A,B). At this time point, ITGB3 expression was below the determined threshold of 60% (Supplementary Figures S3A,B). Closer monitoring of ITGB3 expression in DicloDILI1 and DicloDILI2 over a time period revealed that ITGB3 correlated inversely with liver enzymes (ALT and AST) and thus, with patient recovery (Supplementary Figures S3A,B). These results suggest that ITGB3 might also be used to monitor disease progression and possibly support decision making for the need of medical interventions.

**FIGURE 4:**
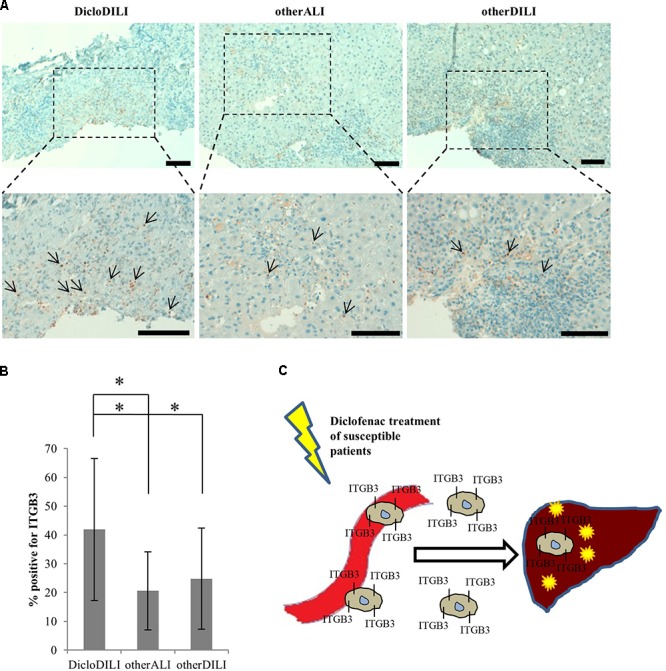
Immunohistochemical staining of liver biopsies with antibody against ITGB3. **(A)** Representative brightfield images of ITGB3 expression in liver biopsies from the different patients (10× and 20×). *Arrows* indicate ITGB3-positive infiltrations. Diffuse *brown* areas represent unspecific staining. Scale bar: 100 μm. **(B)** Relative quantification of ITGB3-positive infiltrations at inflammation sites (*n* = 18 for DicloDILI2, *n* = 17 for other ALI, and *n* = 24 for other DILI. ^∗^*p* < 0.05, ^∗∗^*p* < 0.005, whereas *n* represents the number of inflammatory sites per biopsy). **(C)** Schematic representation of the role of ITGB3 in Diclofenac-DILI. After initial liver injury by Diclofenac, ITGB3-positive cells may be specifically recruited into the liver of susceptible patients and contribute to inflammation and liver damage.

Finally, we searched for an explanation why ITGB3 is upregulated in Diclofenac-treated MH cells from DicloDILI patients, but downregulated in whole blood of the same subject population. We postulated that ITGB3-positive cells would be recruited from the peripheral blood into the liver during Diclofenac-induced liver injury. To prove this theory, we performed immunohistochemical staining for ITGB3 in liver biopsies of DicloDILI2, one other ALI and one other DILI patient. We observed an enhanced expression of ITGB3 for DicloDILI2 compared to the other two samples, predominantly at inflammation sites (**Figure 4A**). Quantification of ITGB3 expression at these sites revealed significant differences between DicloDILI2 and other ALI/other DILI (**Figure 4B**).

## Discussion

The need for specific DILI biomarkers is an international acknowledged issue targeted by the EMA through the IMI projects SAFE-T^1^ and MIP-DILI^2^. Moreover, the United States FDA encourages these programs and has issued a letter of support for the further development and use of the biomarkers identified by the SAFE-T Consortium (Food Drug Administration, 2016). Nevertheless, up to date, DILI biomarker candidates are either not liver specific, or they cannot differentiate between the causes for liver damage and ultimately between the responsible drugs (Andrade et al., 2009). To our knowledge, we are the first to report on a combined technique which uses proteomics analysis of MH cells to identify drug-specific biomarkers. Through analysis of protein expression profiles of MH cells from different donors, we found that patients suffering from Diclofenac-DILI cluster away from healthy controls and patients with liver injury due to other causes. Thus, differences in baseline protein expression might reflect predisposition to develop Diclofenac-DILI, which is known to affect only susceptible patients (Boelsterli, 2003). Moreover, Diclofenac-treatment of MH cells from DicloDILI patients lead to upregulation of a significant amount of proteins. Pathway enrichment analysis revealed that many of these proteins are involved in immune response and metabolism by cytochrome P450 enzymes. This is in line with existing data, showing that both mechanisms contribute to hepatocellular injury by Diclofenac (Boelsterli, 2003). The same data set also showed enrichment in cell adhesion molecules, such as ITGB3. This suggests that ITGB3 plays an important role in inflammatory response and liver regeneration upon injury through Diclofenac-treatment. In support of this theory, our preliminary results showed ITGB3 accumulation at inflammatory sites in the liver of a DicloDILI patient, compared to one other ALI and one other DILI subject. This indicates that ITGB3-expressing cells might be recruited into the liver upon injury, which would also explain their decrease in peripheral blood (**Figure 4C**).

A major challenge in the search for drug-specific DILI-biomarkers is the rare frequency of events. Even in large registries, iDILI cases adjudicated to the same drug rarely exceed 10 or 20 individuals. Thus, even a few false positives can cause significant dilution and lead to disappointing results. In this proof-of-concept study we could show that the combination of novel techniques for diagnosis and sample analysis can help to overcome major challenges in the iDILI setting: Even few test subjects allow the identification of promising novel markers. Taken altogether, this study provides evidence for ITGB3 as potential biomarker for Diclofenac-induced DILI. Although limited by a small sample cohort, ITGB3 meets all of the criteria defined by EMA for DILI biomarkers (European Medicines Agency, 2016). Furthermore, investigations to support our findings are ongoing: currently, we could confirm the ITGB3 decrease in blood in three cases with DicloDILI. EMA requires that the DILI biomarker provides an early or earlier diagnosis compared to current standards. ITGB3 can be used as an early diagnostic marker, which specifically detects Diclofenac-DILI from a small blood sample. ITGB3 can be as easily assessed as liver enzymes, and in contrast, is able to identify the cause of the liver damage. According to EMA, a DILI biomarker should also have the ability to predict DILI outcome and monitor disease progression. In this regard, ITGB3 expression correlated inversely with progression/remission in two individual patients. Additionally, the patient displaying the lowest ITGB3 values was the one requiring a liver transplant, suggesting that ITGB3 might also be used for outcome prediction. The last criterion of EMA refers to the ability of the biomarker to differentiate between adaptors and patients which incur upon drug treatment. ITGB3 was found to be upregulated only in Diclofenac-treated MH cells from DicloDILI patients and not in healthy subjects exposed to Diclofenac. Nevertheless, future investigations for the analysis of initial liver enzyme elevations, followed by ALI or adaption and correlations with ITGB3 expression are required. As a current context of use we propose measurement of ITGB3 in whole blood derived from patients who present with ALI and a history of Diclofenac intake. If ITGB3 is decreased, this strongly points toward the diagnosis DILI by Diclofenac. Further studies of ITGB3 expression and regulation shall provide valuable insights into cellular mechanisms underlying Diclofenac-DILI, thereby addressing a pressing challenge in the field of iDILI characterization and prediction (Funk and Roth, 2017).

## Ethics Statement

This study was conducted in accordance with the ethical standards of the Declaration of Helsinki (1964) and was approved by the local ethics committee (Project No. 055-13; ClinicalTrials.gov Identifier NCT02353455). All participating subjects have signed the informed consent form prior to their inclusion in the study.

## Author Contributions

DD, AB, and AG conceived and designed the experiments. GP and NK performed proteomics measurements and analysis. DD performed MH testing, FACS experiments, and data analysis with AB. HB performed immunohistological stainings and analysis. DD and AB wrote the paper. AG critically revised and approved the manuscript.

## Conflict of Interest Statement

AB: stockholder MetaHeps GmbH and owner of IP. DD: employee MetaHeps GmbH. AG: stockholder MetaHeps GmbH and owner of IP. GP and NK: founders of PreOmics GmbH. HB: nothing to disclose.

## Footnotes

1 https://www.imi.europa.eu/projects-results/project-factsheets/safe-t

2 https://www.imi.europa.eu/projects-results/project-factsheets/mip-dili

## Abbreviations

ALI: acute liver injury
ALT: alanine aminotransferase
APAP: acetaminophen
AST: aspartate aminotransferase
BSA: bovine serum albumin
ITGB3: integrin beta 3
DILI: drug-induced liver injury
DILIN: drug-induced liver injury network
DMSO: dimethylsulfoxide
EDTA: ethylenediaminetetraacetic acid
EMA: European Medicines Agency
FDA: Food and Drug Administration
HMGB1: high-mobility group box-1
iDILI: idiosyncratic drug-induced liver injury
IMI: innovative medicines initiative
Lys-C: endoproteinase Lys-C
MCSFR1: macrophage stimulating factor receptor 1
MH cells: monocyte-derived hepatocyte-like cells
MS: mass spectrometry
NSAID: nonsteroidal anti-inflammatory drug
PCA: principal component analysis
ROC: receiver operating characteristic
RUCAM: Roussel Uclaf Causality Assessment Method
SDC: sodium deoxycholate

## References

[B1] Altman R. Bosch B. Brune K. Patrignani P. Young C. (2015). Advances in NSAID development: evolution of diclofenac products using pharmaceutical technology. *Drugs* 75 859–877. 10.1007/s40265-015-0392-z 25963327 PMC4445819

[B2] Anderson N. Borlak J. (2011). Correlation versus Causation? Pharmacovigilance of the analgesic flupirtine exemplifies the need for refined spontaneous ADR reporting. *PLoS One* 6:e25221. 10.1371/journal.pone.0025221 22022383 PMC3191146

[B3] Andrade R. J. Robles M. Lucena M. I. (2009). Rechallenge in drug-induced liver injury: the attractive hazard. *Expert Opin. Drug Saf.* 8 709–714. 10.1517/14740330903397378 19968572

[B4] Atienzar F. A. Blomme E. A. Chen M. Hewitt P. Kenna J. G. Labbe G. (2016). Key challenges and opportunities associated with the use of in vitro models to detect human DILI: integrated risk assessment and mitigation plans. *Biomed Res. Int.* 2016:9737920. 10.1155/2016/9737920 27689095 PMC5027328

[B5] Bell L. N. Chalasani N. (2009). Epidemiology of idiosyncratic drug-induced liver injury. *Semin. Liver Dis.* 29 337–347. 10.1055/s-0029-1240002 19826967 PMC2903197

[B6] Benesic A. Gerbes A. L. (2018). Herbal tea and liver injury – Tea extract or comedication can make a difference. *J. Hepatol.* 10.1016/j.jhep.2018.03.033 [Epub ahead of print]. 29776714

[B7] Benesic A. Leitl A. Gerbes A. L. (2016). Monocyte-derived hepatocyte-like cells for causality assessment of idiosyncratic drug-induced liver injury. *Gut* 65 1555–1563. 10.1136/gutjnl-2015-309528 26045135

[B8] Benesic A. Rahm N. L. Ernst S. Gerbes A. L. (2012). Human monocyte-derived cells with individual hepatocyte characteristics: a novel tool for personalized in vitro studies. *Lab. Invest.* 92 926–936. 10.1038/labinvest.2012.64 22469698

[B9] Benesic A. Rotter I. Dragoi D. Weber S. Buchholtz M.-L. Gerbes A. L. (2018). Development and validation of a test to identify drugs that cause idiosyncratic drug-induced liver injury. *Clin. Gastr. Hepatol.* 10.1016/j.cgh.2018.04.049 [Epub ahead of print]. 29723689

[B10] Björnsson E. S. (2016). Hepatotoxicity by drugs: the most common implicated agents. *Int. J. Mol. Sci.* 17:224. 10.3390/ijms17020224 26861310 PMC4783956

[B11] Boelsterli U. A. (2003). Diclofenac-induced liver injury: a paradigm of idiosyncratic drug toxicity. *Toxicol. Appl. Pharmacol.* 192 307–322. 10.1016/S0041-008X(03)00368-5 14575648

[B12] Cox J. Hein M. Y. Luber C. A. Paron I. Nagaraj N. Mann M. (2014). Accurate proteome-wide label-free quantification by delayed normalization and maximal peptide ratio extraction, termed MaxLFQ. *Mol. Cell. Proteomics* 13 2513–2526. 10.1074/mcp.M113.031591 24942700 PMC4159666

[B13] Cox J. Mann M. (2008). MaxQuant enables high peptide identification rates, individualized p.p.b.-range mass accuracies and proteome-wide protein quantification. *Nat. Biotechnol.* 26 1367–1372. 10.1038/nbt.1511 19029910

[B14] Cox J. Neuhauser N. Michalski A. Scheltema R. A. Olsen J. V. (2011). Andromeda: a peptide search engine integrated into the MaxQuant environment. *J. Proteome Res.* 10 1794–1805. 10.1021/pr101065j 21254760

[B15] Danan G. Teschke R. (2016). RUCAM in drug and herb induced liver injury: the update. *Int. J. Mol. Sci.* 17:E14. 10.3390/ijms17010014 26712744 PMC4730261

[B16] European Medicines Agency (2016). *Letter of Support for Drug-Induced Liver Injury (DILI) Biomarker*. Available at: http://www.ema.europa.eu/docs/en_GB/document_library/Other/2016/09/WC500213479.pdf

[B17] Fontana R. J. Watkins P. B. Bonkovsky H. L. Chalasani N. Davern T. Serrano J. (2009). Drug-Induced Liver Injury Network (DILIN) Prospective Study. *Drug Saf.* 32 55–68. 10.2165/00002018-200932010-00005 19132805 PMC3637941

[B18] Food Drug Administration (2016). *Letter of Support for Drug-Induced Liver Injury (DJU) Biomarker(s).* Available at: https://www.fda.gov/downloads/Drugs/DevelopmentApprovalProcess/UCM514812.pdf

[B19] Funk C. Roth A. (2017). Current limitations and future opportunities for prediction of DILI from in vitro. *Arch. Toxicol.* 91 131–142. 10.1007/s00204-016-1874-9 27766365

[B20] Harris E. S. McIntyre T. M. Prescott S. M. Zimmerman G. A. (2000). The leukocyte integrins. *J. Biol. Chem.* 275 23409–23412. 10.1074/jbc.R000004200 10801898

[B21] Hayashi P. H. (2016). Drug-induced liver injury network causality assessment: criteria and experience in the United States. *Int. J. Mol. Sci.* 17:201. 10.3390/ijms17020201 26861284 PMC4783935

[B22] Kulak N. A. Pichler G. Paron I. Nagaraj N. Mann M. (2014). Minimal, encapsulated proteomic-sample processing applied to copy-number estimation in eukaryotic cells. *Nat. Methods* 11 319–324. 10.1038/nmeth.2834 24487582

[B23] Kullak-Ublick G. A. Andrade R. J. Merz M. End P. Benesic A. Gerbes A. L. (2017). Drug-induced liver injury: recent advances in diagnosis and risk assessment. *Gut* 66 1154–1164. 10.1136/gutjnl-2016-313369 28341748 PMC5532458

[B24] Luscinskas F. W. Lawler J. (1994). Integrins as dynamic regulators of vascular function. *FASEB J.* 8 929–938. 10.1096/fasebj.8.12.7522194 7522194

[B25] Robles-Díaz M. Medina-Caliz I. Stephens C. Andrade R. J. Lucena M. I. (2016). Biomarkers in DILI: one more step forward. *Front. Pharmacol.* 7:267. 10.3389/fphar.2016.00267 27597831 PMC4992729

[B26] Scheltema R. A. Mann M. (2012). SprayQc: a real-time LC-MS/MS quality monitoring system to maximize uptime using off the shelf components. *J. Proteome Res.* 11 3458–3466. 10.1021/pr201219e 22515319

[B27] Schmeltzer P. A. Kosinski A. S. Kleiner D. E. Hoofnagle H. J. Stolz A. Fontana R. J. (2016). Liver injury from nonsteroidal anti-inflammatory drugs in the United States. *Liver Int.* 36 603–609. 10.1111/liv.13032 26601797 PMC5035108

[B28] Singer J. B. Lewitzky S. Leroy E. Yang F. Zhao X. Klickstein L. (2010). A genome-wide study identifies HLA alleles associated with lumiracoxib-related liver injury. *Nat. Genet.* 42 711–714. 10.1038/ng.632 20639878

[B29] Spraggs C. F. Budde L. R. Briley L. P. Bing N. Cox C. J. King K. S. (2011). HLA-DQA1^∗^02:01 is a major risk factor for lapatinib-induced hepatotoxicity in women with advanced breast cancer. *J. Clin. Oncol.* 29 667–673. 10.1200/JCO.2010.31.3197 21245432

[B30] Teschke R. Eickhoff A. (2016). The honolulu liver disease cluster at the medical center: its mysteries and challenges. *Int. J. Mol. Sci.* 17:476. 10.3390/ijms17040476 27043544 PMC4848932

[B31] Teschke R. Frenzel C. Wolff A. Eickhoff A. Schulze J. (2014). Drug induced liver injury: accuracy of diagnosis in published reports. *Ann. Hepatol.* 13 248–255. 24552867

[B32] Teschke R. Schulze J. Eickhoff A. Danan G. (2017). Drug induced liver injury: can biomarkers assist RUCAM in causality assessment? *Int. J. Mol. Sci.* 18:803. 10.3390/ijms18040803 28398242 PMC5412387

[B33] Tyanova S. Temu T. Sinitcyn P. Carlson A. Hein M. Geiger T. (2016). The Perseus computational platform for comprehensive analysis of (prote)omics data. *Nat. Methods* 13 731–740. 10.1038/nmeth.3901 27348712

