# Supplementary material for: Proteomics Analysis of Monocyte-Derived Hepatocyte-Like Cells Identifies Integrin Beta 3 as a Specific Biomarker for Drug-Induced Liver Injury by Diclofenac
Source: Front Pharmacol. 2018 Jul 4;9:699. doi: 10.3389/fphar.2018.00699 (PMC6039575; doi:10.3389/fphar.2018.00699)
Supplement: Supplementary file 1 [file Data_Sheet_1.pdf]

Diagnosis	iDILI				other ALI	
**Causality likelihood**	definite	highly likely		probable	possible	unlikely
**Other causes of liver injury**	excluded	excluded		unlikely	probable	highly probable
**Drug signature**	typical	typical	atypical	atypical	compatible	atypical
**Co-medication**	none	compatible signature	none	compatible signature	any	any
**Re-challenge**	positive with single agent*	positive with multiple agents**		not done	not done	negative

Supplementary Table S1: Template for causality likelihood used for iDILI diagnosis and drug causality. For each case diagnosis was made by assessing each drug involved. The highest causality likelihood equals the case likelihood and therefore diagnosis of iDILI or other ALI (acute liver injury). *positive re-challenge with a single agent proved the respective drug as definite. **Positive re-challenge with multiple drugs resulted in “highly likely” for the drugs with positive re-challenge.

characteristics	DicloDILI	Diclo_tolerator	Diclo_otherDILI	Diclo_otherALI	Control_healthy	Control_otherDILI	Control_otherALI	P DicloDILI vs other goups
total N	4	3	2	3	3	4	3	-
female N	1	2	0	0	1	3	1	0,535
female%	0,25	0,67	0	0	0,33	0,75	0,33	-
caucasian N	3	3	2	2	3	4	3	0,338
caucasian%	0,75	1	1	0,67	1	1	1	-
Age (Median/Range)	52,5 (15-59)	37 (29-40)	38,5 (35-42)	39 (26-73)	26 (24-49)	68 (52-79)	52 (40-70)	0,967
Hepatocellular pattern N	3	na	2	1	na	2	0	0,279
Hepatocellular%	0,75	na	1	0,33	na	0,5	0	-
Severity (Median/Range)	1,5 (1-4)	na	2 (1-3)	2 (2-3)	na	2 (1-4)	1 (1-2)	0,862
Acute liver failure N	1	na	1	1	na	1	0	0,603
Acute liver failure%	0,25	na	0,5	0,33	na	0,25	0	-
RUCAM Diclo (Median/Range)	8 (7-9)	na	4,5 (4-5)	4 (-1-4)	na	na	na	0,016*
RUCAM other drug (Median/Range)	6,5 (5-8)	na	7,5 (7-8)	4 (-1-5)	na	6,5 (6-7)	5 (2-5)	0,379
MH positive Diclo	4	na	0	0	na	0	0	-
MH positive other drug	0	na	2	0	na	4	0	-

Supplementary Table S2: Characteristics of study subjects and, where applicable detail on liver injury. Severity was scored as described in Ref. 15. *p<0.05 vs all other groups.

Subject ID	RUCAM Diclo	other drug	RUCAM other drug	Group based on causality assessment (ALI diagnosis)	Investigations leading to diagnosis	Underlying disease (drug indication)	gender	age	ethnicity	severity	pattern	acute liver failure	latency (days)	dose (mg)
DicloDILI1	8	progesterone	5	DicloDILI	Exclusion of other causes, Diclofenac with compatible time to onset and pattern	none (headache)	female	50	hispanic	4	Hepato-cellular	yes	87	150
DicloDILI2	9	Phenprocoumon	8	DicloDILI	Exclusion of other causes, Diclofenac with compatible time to onset and pattern	arterial hypertension (back pain)	male	59	caucasian	2	Hepato-cellular	no	32	150
DicloDILI3	8	Pantoprazole	8	DicloDILI	Exclusion of other causes, Diclofenac with compatible time to onset and pattern	none (dicus prolaps)	male	55	caucasian	1	Hepato-cellular	no	68	150
DicloDILI4	7	Cefaclor	5	DicloDILI	Exclusion of other causes, Diclofenac with compatible time to onset and pattern	heart insufficiency due to perinatal myocarditis (flu symptoms)	male	15	caucasian	1	Chole-static	no	7	75
Diclo_ tolerator1	N/A	N/A	N/A	Diclo_tolerator	N/A	N/A	male	37	caucasian	N/A	N/A	N/A	N/A	N/A
Diclo_ tolerator2	N/A	N/A	N/A	Diclo_tolerator	N/A	N/A	female	40	caucasian	N/A	N/A	N/A	N/A	N/A
Diclo_ tolerator3	N/A	N/A	N/A	Diclo_tolerator	N/A	N/A	female	29	caucasian	N/A	N/A	N/A	N/A	N/A
Diclo_ otherDILI1	4	Carbi-mazole	7	Diclo_otherDILI	Exclusion of other causes, Carbimazole with compatible time to onset and pattern	subarachnoid hemorrhage (hyperthyreosis)	male	42	caucasian	1	Hepato-cellular	no	19 (Carbi-mazole); 1 (Diclo-fenac)	10 (Carbi-mazole); 150 (Diclo-fenac)
Diclo_ otherDILI2	5	Meta-mizole	8	Diclo_otherDILI	Exclusion of other causes, Metamizole with compatible time to onset and pattern	impingement syndrom (post shoulder surgery)	male	35	caucasian	3	Hepato-cellular	yes	68 (Metami-zole); 311 (Diclo-fenac)	1500 (Metamizole); 150 (Diclo-fenac)
Diclo_ otherALI1	4	Cipro-floxacin	4	Diclo_otherALI (Adult Still's Disease)	Laboratory, symptoms, serology	none (arthralgias, fever)	male	73	caucasian	2	Chole-static	no	10 (Diclo-fenac)	150 (Diclo-fenac)
Diclo_ otherALI2	-1	Amoxi-cillin /Clavulanate	-1	Diclo_otherALI (acute Hepatitis A)	Serology, viral load	none (fever, abdominal pain)	male	26	arabic	3	Hepato-cellular	yes	23 (Diclo-fenac)	150 (Diclo-fenac)
Diclo_ otherALI3	4	Meta-mizole	5	Diclo_otherALI (Benign recurrent intrahepatic cholestasis)	History, genetics	cutaneous abscess post surgery	male	39	caucasian	2	Chole-static	no	21 (Diclo-fenac)	150 (Diclo-fenac)
Control_ healthy1	N/A	N/A	N/A	Control_healthy	N/A	N/A	male	26	caucasian	N/A	N/A	N/A	N/A	N/A
Control_ healthy2	N/A	N/A	N/A	Control_healthy	N/A	N/A	male	24	caucasian	N/A	N/A	N/A	N/A	N/A
Control_ healthy3	N/A	N/A	N/A	Control_healthy	N/A	N/A	female	49	caucasian	N/A	N/A	N/A	N/A	N/A
Control_ otherDILI1	N/A	Dabi-gatran	6	Control_otherDILI	Exclusion of other causes, Dabigatran with compatible time to onset and pattern	atrial fibrillation	female	79	caucasian	1	Chole-static	no	141 (Dabi-gatran)	15 (Dabi-gatran)
Control_ otherDILI2	N/A	Amoxi-cillin /Clavu-lanate	7	Control_otherDILI	Exclusion of other causes, Amoxicillin/Clavulanate with compatible time to onset and pattern	dental surgery	female	52	caucasian	2	Chole-static	no	19 (Amox-Clav)	2000 (Amox-Clav)
Control_ otherDILI3	N/A	Pirfeni-done	6	Control_otherDILI	Exclusion of other causes, Pirfenidone with compatible time to onset and pattern	idiopathic pulmonary fibrosis	male	76	caucasian	4	Hepato-cellular	yes	442 (Pir-fenidone)	800 (Pir-feni-done)
Control_ otherDILI4	N/A	Amoxi-cillin /Clavu-lanate	7	Control_otherDILI	Exclusion of other causes, Amoxicillin/Clavulanate with compatible time to onset and pattern	restless legs (pneumonia)	female	60	caucasian	2	Hepato-cellular	no	5 (Amox-Clav)	2000 (Amox-Clav)
Control_ otherALI1	N/A	Amoxi-cillin /Clavu-lanate	5	Control_otherALI (alcohol)	History, laboratory	brest cancer (post surgery)	female	52	caucasian	1	Chole-static	no	5 (Amox-Clav)	3000 (Amox-Clav)
Control_ otherALI2	N/A	Mero-penem	5	Control_otherALI (secondary sclerosing cholangitis)	Imaging, course of disease	subarachnoid hemorrhage (post surgery)	male	70	caucasian	1	Chole-static	no	25 (Mero-penem)	1500 (Mero-penem)
Control_ otherALI3	N/A	Moxi-floxacin	2	Control_otherALI (Vasculitis)	Serology, clinical symptoms, organ involvement	Vasculitis (Fever and malaise)	male	40	caucasian	2	Chole-static	no	3 (Moxi-floxacin)	400 (Moxi-floxacin)

Supplementary Table S3: Details on study subject diagnosis, comorbidities and comedications.

Name	No. of proteins	Enrichment	P-value
Spliceosome	25	1,9	0,0003
Protein processing in endoplasmic reticulum	19	1,4	0,0385
Antigen processing and presentation	9	1,7	0,0366
Cell adhesion molecules (CAMs)	8	1,7	0,0481
Metabolism of xenobiotics by cytochrome P450	6	2,3	0,0242

Supplementary Table S4: Pathways significantly enriched in the protein cluster predominantly upregulated in DicloDILI (dotted rectangle in Fig. 2a).

Protein name	Gene name	Cellular localization	Relative change in expression: Diclo vs. vehicle control						
			Diclo DILI	Diclo_ tolerator	Diclo_ other DILI	Diclo_ other ALI	Control_ healthy	Control_ other DILI	Control_ otherALI
Tubulin beta-1 chain	TUBB1	intracellular	32,723	0,446	0,513	1,222	0,671	0,782	0,938
Fatty acyl-CoA reductase 1	FAR1	intracellular	7,800	0,852	0,951	1,678	0,969	1,092	0,486
Nicotinamide phosphoribosyltransferase	NAMPT	intracellular	6,974	1,105	1,033	1,075	1,093	1,365	1,138
EH domain-containing protein 3	EHD3	intracellular	5,713	0,811	1,498	0,827	1,052	0,601	1,437
GPI transamidase component PIG-T	PIGT	intracellular	5,039	0,835	1,123	0,908	0,941	0,992	1,004
Pseudouridine-5-monophosphatase	HDHD1	intracellular	4,790	1,080	1,093	0,764	1,491	1,097	1,076
Misshapen-like kinase 1	MINK1	intracellular	4,600	0,918	0,931	1,193	0,967	0,964	0,661
Leukocyte elastase inhibitor	SERPINB1	intracellular	4,438	0,964	0,877	1,001	1,027	1,066	1,116
Integrin beta 3	ITGB3	membrane	4,204	1,384	0,684	0,867	0,622	1,304	0,730
Receptor expression-enhancing protein 3	REEP3	intracellular	4,195	1,061	1,070	1,341	0,849	0,656	1,042

Supplementary Table S5: List of first ten upregulated proteins in Diclofenac-treated MH cells from DicloDILI subjects, compared to vehicle control.

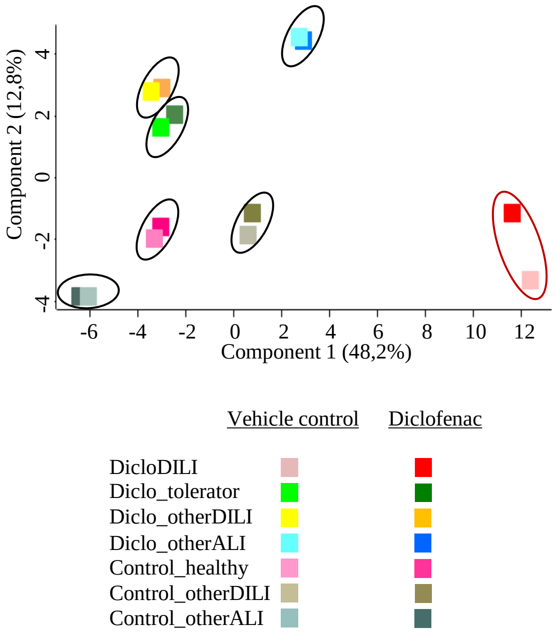

Supplementary Figure S1: Principal component analysis of protein expression in MH cells from the different subject groups. Circles indicate samples which belong to the same subject group and were treated with DMSO (vehicle control) or Diclofenac.

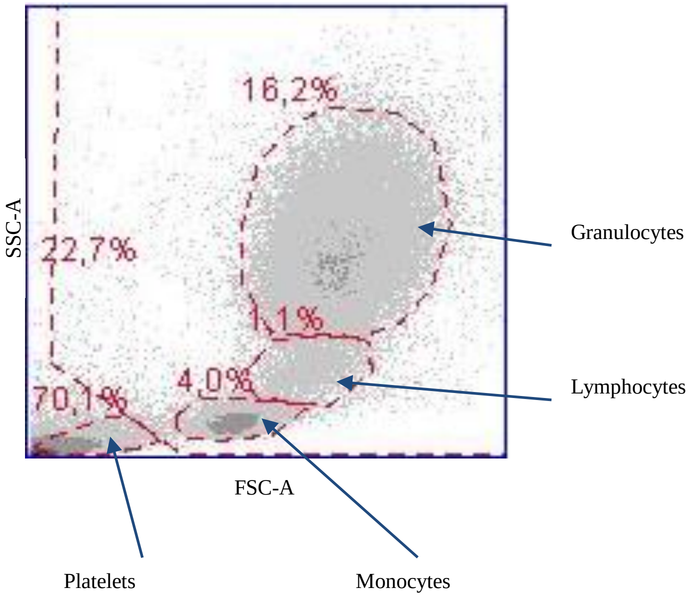

Supplementary Figure S2: Gating strategy for the analysis of erythrocyte-lysed whole blood by flow cytometry. Representative density plot (forward vs side scatter area), showing the different blood cell populations.

(A)

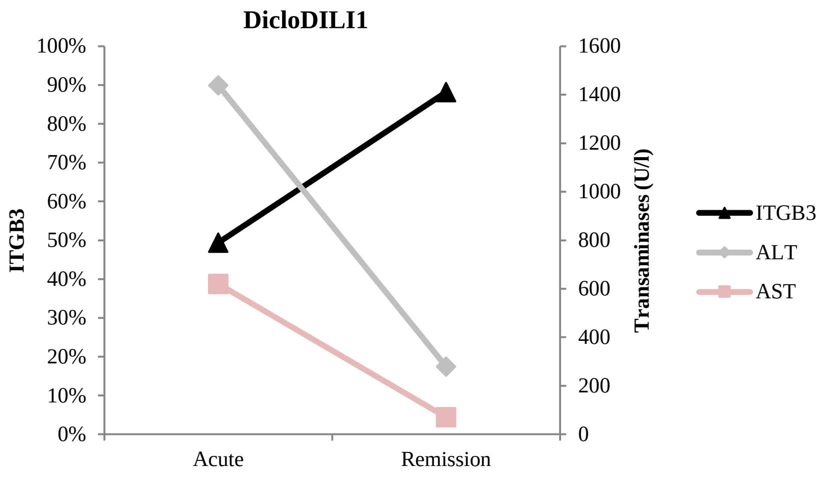

(B)

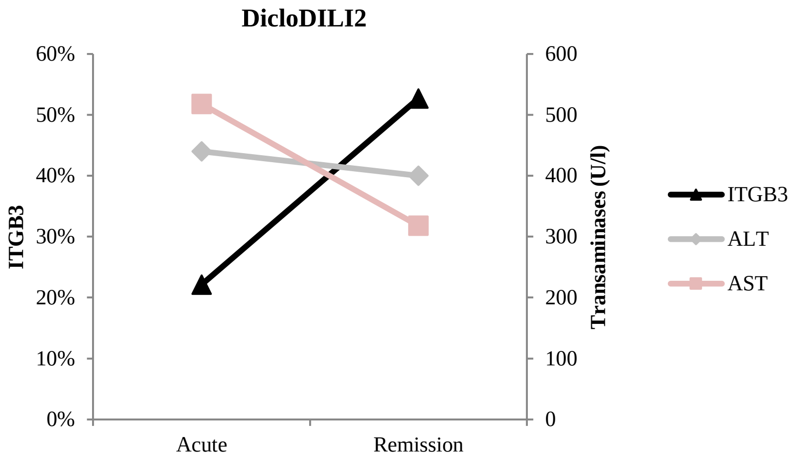

Supplementary Figure S3: Evolution of ITGB3 values and liver transaminases (ALT, AST) in DicloDILI1 **(A)** and DicloDILI2 **(B)** from the acute phase of the disease to remission. ITGB3 correlated inversely to ALT and AST in both subjects.
